# Injurious Effects of Curcumin on Maturation of Mouse Oocytes, Fertilization and Fetal Development via Apoptosis

**DOI:** 10.3390/ijms13044655

**Published:** 2012-04-12

**Authors:** Chia-Chi Chen, Wen-Hsiung Chan

**Affiliations:** Department of Bioscience Technology and Center for Nanotechnology, Chung Yuan Christian University, Chung Li 32023, Taiwan; E-Mail: cha_chi_chen@yahoo.com.tw

**Keywords:** curcumin, apoptosis, oocyte maturation, embryonic development

## Abstract

Curcumin, a common dietary pigment and spice, is a hydrophobic polyphenol derived from the rhizome of the herb *Curcuma longa*. Previously, we reported a cytotoxic effect of curcumin on mouse embryonic stem cells and blastocysts and its association with defects in subsequent development. In the present study, we further investigated the effects of curcumin on oocyte maturation and subsequent pre- and post-implantation development, both *in vitro* and *in vivo*. Notably, curcumin induced a significant reduction in the rate of oocyte maturation, fertilization, and *in vitro* embryonic development. Treatment of oocytes with curcumin during *in vitro* maturation (IVM) led to increased resorption of postimplantation embryos and decreased fetal weight. Experiments with an *in vivo* mouse model disclosed that consumption of drinking water containing 40 μM curcumin led to decreased oocyte maturation and *in vitro* fertilization as well as early embryonic developmental injury. Finally, pretreatment with a caspase-3-specific inhibitor effectively prevented curcumin-triggered injury effects, suggesting that embryo impairment by curcumin occurs mainly via a caspase-dependent apoptotic process.

## 1. Introduction

Curcumin is used as a traditional Indian medicine [1] for the treatment of wounds, liver ailments, hepatitis and urinary tract diseases, as well as a cosmetic [2]. Curcumin exerts a wide range of pharmacological effects, including anti-inflammatory, anti-carcinogenic, hypocholesterolemic and anti-infection activities [3–8]. As a potential antioxidant, curcumin displays anti-proliferative and anti-carcinogenic properties in a variety of cell lines and animals [8–12]. Moreover, the efficacy of curcumin in various diseases, including cancer, is well established [13]. Recent studies have shown that the anti-tumor activity of curcumin is attributed to its ability to induce apoptosis via caspase-3 activation [14,15]. Moreover, various *in vivo* animal assay models and human studies have confirmed that dietary curcumin is extremely safe and does not exert hazardous effects, even at high doses [16–19]. For example, three separate phase I clinical trials have demonstrated that dietary curcumin administered at doses as high as 12 g per day is well tolerated in human [18–20]. Owing to its high pharmacological safety and efficacy, curcumin is a potential therapeutic candidate for the treatment and prevention of a wide range of human diseases.

In a recent study, we showed that curcumin inhibits methylglyoxal-induced reactive oxygen species (ROS) generation and various apoptotic biochemical events in embryonic stem cells and blastocysts isolated from pregnant mice [21]. Another investigation by our group focusing on the possible effects of curcumin on ROS generation, intracellular adenosine triphosphate (ATP) levels and cell death mode in osteoblast cells revealed that curcumin induces apoptosis or necrosis in a dose-dependent manner [15]. A more recent study by our laboratory showed that *in vitro* exposure of blastocysts to curcumin triggers apoptosis and retards early postimplantation development after transfer to host mice. In addition, curcumin induces apoptotic injury effects on mouse blastocysts through ROS generation and further promotes mitochondria-dependent apoptotic signaling processes to impair sequent embryonic development [22]. However, while multiple biological functions have been identified for curcumin, the ambiguous issue of its activity as an apoptotic inducer or inhibitor and the precise molecular mechanisms underlying these actions are yet to be fully determined. To date, no studies have investigated the potential of curcumin as a cytotoxic agent against oocyte maturation, fertilization and sequent embryonic development.

Oocyte viability is affected by the microenvironment during growth and maturation. Heat stress, oxygen concentration and glucose content are key determinants of oocyte viability [23–25]. A number of researchers have investigated the influence of environmental biological toxins on oocyte maturation *in vivo* and *in vitro*. During normal embryogenesis, apoptosis (a unique morphological pattern of cell death) functions to remove abnormal or redundant cells in preimplantation embryos [26,27]. However, apoptotic processes do not occur prior to the blastocyst stage during normal mouse embryonic development [28], and induction of cell death during oocyte maturation and early stages of embryogenesis (*i.e.*, via exposure to a teratogen) leads to embryonic developmental injury [21,24,29–31]. Previous studies by our group have demonstrated that curcumin promotes cell apoptosis and developmental injury in blastocyst stage embryos [22]. However, the influence of curcumin on early stage embryogenesis processes, such as oocyte maturation, fertilization, and sequential embryo development from zygotes, is currently unclear. Here, we further investigated whether curcumin exerts a hazardous effect on oocyte development by incubating oocytes with the compound for 24 h and comparing sequential development with that of oocytes under curcumin-free conditions. We aimed to determine whether short-term exposure to curcumin at the oocyte stage has a long-term injurious impact on embryo development. Our results clearly demonstrate that curcumin exposure during the oocyte stage not only inhibits oocyte maturation but also promotes injurious effects on *in vitro* fertilization and embryonic development.

## 2. Results and Discussion

**Figure 1:**
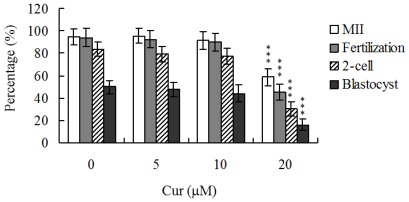
Effects of curcumin on mouse oocyte maturation and embryo development *in vitro*. Oocytes were collected from 21 day-old mice, cultured for 24 h in *in vitro* Maturation (IVM) medium containing curcumin (Cur; 5, 10 or 20 μM), fertilized *in vitro*, and transferred to *in vitro* culture (IVC) medium. Oocyte maturation, *in vitro* fertilization, cleavage and blastocyst development were analyzed. Values are presented as means ± SEM of eight determinations. Data are based on 250–300 samples per group. *** *P* < 0.001 *versus* the untreated control group.

Although recent studies have established that curcumin induces apoptosis and developmental injury in mouse blastocysts [22], its effects on oocyte maturation are currently unclear. Oocyte nuclear maturation status was measured using 12 independent experimental replicates (~220 oocytes per group). The majority of oocytes reached the metaphase II (MII) stage of maturation after IVM (ranging to about 94.7%). A lower maturation rate was observed in the 20 μM curcumin-treated oocyte group (Figure 1). Male pronucleus formation was assessed for the detection of fertilization. Notably, the ability of oocytes to be fertilized by fresh sperm was significantly decreased upon pretreatment with 20 μM curcumin prior to IVM (Figure 1).

We further analyzed in *vitro* embryo development to the two-cell and blastocyst stages. Curcumin (20 μM) pretreatment led to a significant decrease in cleavage of oocytes to the two-cell stage, indicative of an injurious effect (Figure 1). In addition, the number of embryos that cleaved to form blastocysts in 20 μM curcumin-treated group was significantly lower than that in untreated control groups (Figure 1).

**Figure 2:**
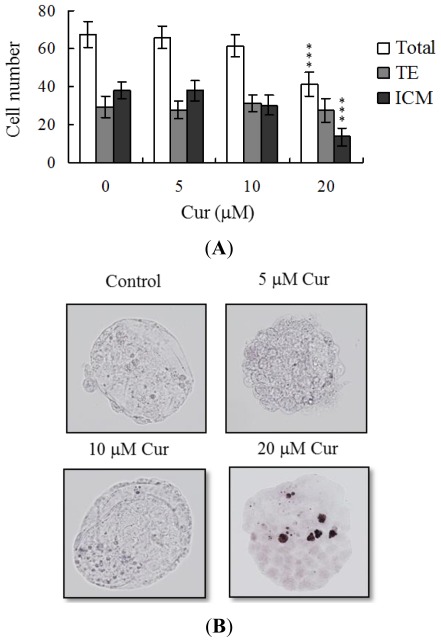

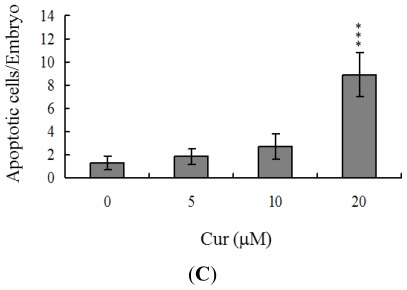
Effects of curcumin on cell number and apoptosis in embryos during IVM of oocytes. Oocytes were cultured for 24 h in IVM medium containing curcumin (Cur; 5, 10 or 20 μM), fertilized *in vitro*, and transferred to *in vitro* culture (IVC) medium for *in vitro* development. (**A**) Cell numbers of total, trophectoderm (TE) lineages and inner cell mass (ICM) were counted in blastocysts; (**B**) Apoptotic cells were examined at the blastocyst stage using TUNEL staining, followed by light microscopy. Positive cells are depicted in black; (**C**) The mean number of apoptotic (TUNEL-positive) cells per blastocyst was calculated. Values are presented as means ± SEM of six replicates. Data are based on at least 300 samples in each group. *** *P* < 0.001 *versus* the untreated control group.

Following curcumin treatment during IVM of oocytes, total blastocyst cell numbers were counted, with a view to establishing its effects on cell proliferation. Differential staining, followed by cell counting, was employed to assess cell proliferation. Significantly lower blastocyst cell numbers were derived from 20 μM curcumin-pretreated oocytes, compared to control oocytes (Figure 2A). Additionally, the number of ICM cells in blastocysts decreased during IVM after pretreatment with 20 μM curcumin (Figure 2A). Curcumin did not affect the number of trophectoderm (TE) cells present in blastocysts (Figure 2A).

Apoptosis of blastocysts derived from 20 μM curcumin-pretreated oocytes was additionally evaluated. TUNEL staining revealed a dose-dependent increase in apoptosis of blastocysts derived from the 20 μM curcumin-pretreated oocyte group (Figure 2B). Further quantitative analysis showed a ~9-fold increase in apoptotic blastocysts derived from 20 μM curcumin-pretreated oocytes, compared to the control group (Figure 2C).

**Figure 3:**
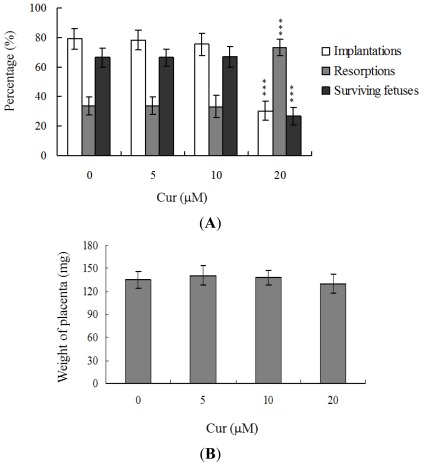

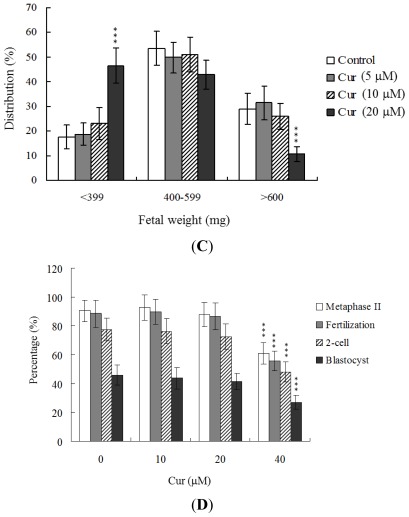
Effects of curcumin treatment or dietary curcumin on embryo development during oocyte IVM. Oocytes were cultured for 24 h in IVM medium containing curcumin (Cur; 5, 10 or 20 μM), fertilized *in vitro*, and transferred to *in vitro* culture medium for development. (**A**) Implantation, resorption and surviving fetuses were analyzed, as described in Materials and Methods. The implantation percentage represents the number of implantations per number of transferred embryos × 100. The percentage of resorbed or surviving fetuses represents the number of resorptions or surviving fetuses per number of implantations × 100; (**B**) Placental weights of 40 recipient mice were measured; (**C**) Weight distribution of surviving fetuses at day 18 post-coitus. Surviving fetuses were obtained by embryo transfer of control and curcumin-pretreated groups, as described in Materials and Methods (320 total blastocysts across 40 recipients); (**D**) Random female mice (21 day-old) were fed a standard diet and drinking water supplemented with curcumin (10–40 μM) for 5 days or left untreated. Oocytes were collected for *in vitro* maturation, *in vitro* fertilization, cleavage, and blastocyst development analyses. Data are based on at least 400 samples in each group. *** *P* < 0.001 *versus* the curcumin-free group.

Embryos were transferred to 54 recipients per group (8 per horn). A total of 40 recipients were pregnant in at least one horn at day 18. The implantation ratio of blastocysts derived from the oocyte group treated with 20 μM curcumin during IVM was ~30%, which was significantly lower than that observed for control blastocysts (~79%) (Figure 3A).

Embryos that implanted but failed to develop were subsequently resorbed in the uterus. The proportion of implanted embryos that failed to develop normally was significantly higher in the 20 μM curcumin-treated group (~73%), compared to the control group (~33.6%) (Figure 3A). In terms of embryo survival rate (surviving fetuses), 66.4% of the control group survived to post-coitus day 18, compared to only 26.8% of the 20 μM curcumin-treated group (Figure 3A). Interestingly, however, the placental weights of blastocysts derived from 20 μM curcumin-treated oocytes in IVM were not significantly different from those of the control group (Figure 3B). Importantly, fetal weights were lower in the groups treated with 20 μM curcumin, relative to the untreated control group. Furthermore, only 10.7% of fetuses in the 20 μM curcumin-pretreated group weighed over 600 mg, an important indicator of successful embryonic and fetal development, whereas 28.9% of control fetuses exceeded this threshold (Figure 3C). Our findings collectively indicate that exposure of oocytes to curcumin during IVM reduces the potential of postimplantation development.

In view of the injurious effects of curcumin on oocyte maturation and embryo development *in vitro*, we analyzed curcumin activity *in vivo* via intake in an animal model. Female mice were fed a standard diet and drinking water supplemented with curcumin (10–40 μM) for 4 days or left untreated, prior to COC collection. Oocyte maturation status, fertilization rate, and *in vitro* embryo development were evaluated. Dietary curcumin at a concentration of 40 μM induced a significant decrease in oocyte maturation and fertilization, resulting in inhibition of embryonic development from the zygote to blastocyst stage (Figure 3D).

**Figure 4:**
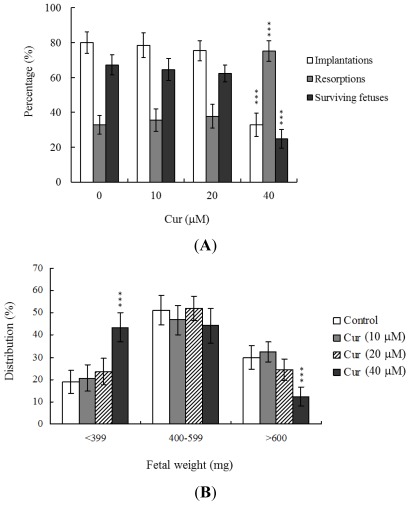
Effects of dietary curcumin on embryo development in mouse blastocysts. Random female mice (21 day-old) were fed a standard diet and drinking water continuously supplemented with curcumin (10–40 μM) or left untreated for 4 days before embryo transfer to the uterus during the experimental period. (**A**) Implantation, resorption and surviving fetuses were analyzed, as described in Materials and Methods. The implantation percentage represents the number of implantations per number of transferred embryos × 100. The percentage of resorbed or surviving fetuses represents the number of resorptions or surviving fetuses per number of implantations × 100; (**B**) Weight distribution of surviving fetuses at day 18 post-coitus. Surviving fetuses were obtained by embryo transfer of control and curcumin intake groups, as described in Materials and Methods (320 total blastocysts across 40 recipients). *** *P* < 0.001 *versus* the curcumin-free group.

To further determine the effects of curcumin on embryo implantation and post-implantation development, we analyzed curcumin activity *in vivo* via intake and transfer of blastocyst stage embryos to the uterus horn using the embryo transfer assay in an animal model. Female mice were fed a standard diet and drinking water continuously supplemented with curcumin (10–40 μM) or left untreated for 4 days before embryo transfer to the uterus during the experimental period. Embryos were transferred to 48 recipients per group (8 per horn). A total of 40 recipients were pregnant at day 18. Notably, the implantation ratio of blastocysts in the 40 μM curcumin-intake group was significantly lower than that of blastocysts in the curcumin-free control group (Figure 4A). Moreover, the 40 μM curcumin intake group displayed a higher overall resorption rate (Figure 4A) and the embryo survival rate was markedly lower than that of curcumin-free group (Figure 4A). However, the placental weights of blastocysts derived from the curcumin-intake group were not significantly different from those of the control group (data not shown). Finally, fetal weights were lower in the curcumin intake (40 μM) than the untreated control group (Figure 4B). Our results collectively demonstrate that exposure of embryos to 40 μM curcumin reduces the potential of implantation and postimplantation development.

**Figure 5:**
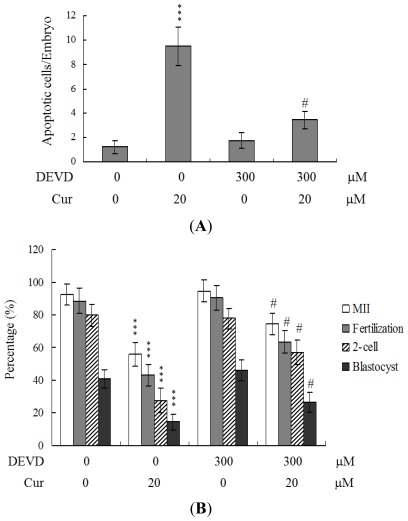

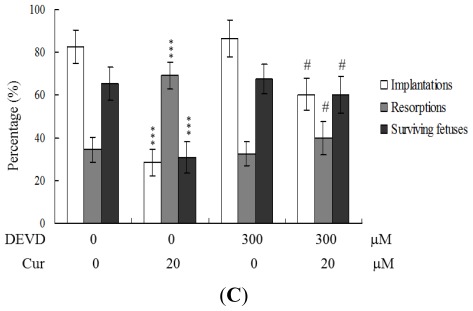
Effects of inhibition of cell apoptosis on embryo development in curcumin treatment during oocyte IVM. Oocytes were collected from 21 day-old mice, cultured for 24 h in IVM medium alone or containing 300 μM Ac-DEVD-cho and 20 μM curcumin (Cur), fertilized *in vitro*, and transferred to *in vitro* culture (IVC) medium for *in vitro* development. (**A**) Apoptotic cells were examined at the blastocyst stage via TUNEL staining; (**B**) Oocyte maturation, *in vitro* fertilization, cleavage and blastocyst development were analyzed; (**C**) Implantation, resorption, and surviving fetuses were analyzed with the embryo transfer assay, as described for Figure 3. Data are based on at least 250 samples in each group. *** *P* < 0.001 *versus* the untreated control group; ^#^ *P* < 0.001 *versus* curcumin-treated group.

To further clarify the regulatory mechanisms underlying curcumin activity, oocytes were pretreated with 300 μM Ac-DEVD-cho, a caspase-3 specific inhibitor, with the aim of preventing curcumin-triggered embryo cell apoptosis during IVM. Pretreatment with the caspase-3 inhibitor effectively prevented apoptosis of cells within the blastocysts derived from the oocyte group pretreated with 20 μM curcumin (Figure 5A). Consistently, the caspase-3 inhibitor blocked curcumin-triggered hazardous effects on oocyte maturation, fertilization rate, and sequential embryo development during IVM (Figure 5B). Treatment with the caspase-3 inhibitor additionally rescued the curcumin-induced reduction in postimplantation development potential by embryo transfer, including embryo implantation and fetal survival rates (Figure 5C). These results strongly indicate that the mechanisms underlying curcumin regulation of oocyte development during IVM involve apoptotic processes.

**Figure 6:**
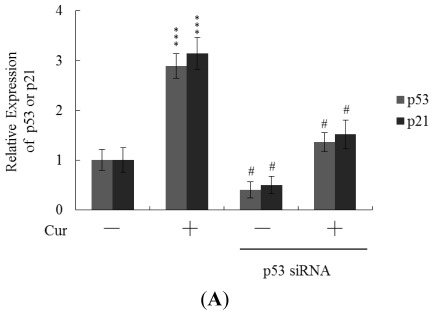

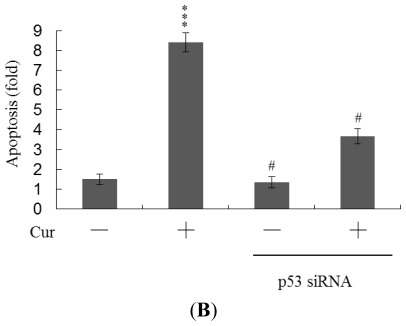
Knockdown of p53 protects blastocyst cells against curcumin-induced apoptosis. Mouse oocytes were transfected with siRNA targeting p53, incubated for 24 h, and treated with 20 μM curcumin (Cur) for a further 24 h. (**A**) The mRNA levels of p53 and p21 were analyzed using real-time PCR; (**B**) Apoptosis was measured as described in Figure 1. *** *P* < 0.001 *versus* the untreated control group; ^#^ *P* < 0.001 *versus* the “curcumin-treated” group.

Real-time RT-PCR revealed that the levels of mRNA encoding p53 and p21 were significantly upregulated in cells of blastocysts from oocytes pretreated with 20 μM curcumin (Figure 6A). To further explore the functions of p53 and p21 in terms of curcumin-induced apoptosis, we used targeted siRNAs to suppress p53 expression in blastocyst cells derived from oocytes. Knockdown with p53 siRNA significantly decreased the levels of mRNA encoding p53 and p21 in blastocyst cells derived from oocytes pretreated with 20 μM curcumin (Figure 6A); this was accompanied by a marked decrease in the extent of curcumin-induced apoptosis (Figure 6B). The results indicate that curcumin upregulates the levels of p53 and p21 in blastocyst cells derived from curcumin-pretreated oocytes; apoptosis is subsequently promoted. Thus, regulation of apoptosis in blastocyst cells derived from curcumin-treated oocytes involves p53-dependent biochemical processes; embryo implantation and fetal survival rates are subsequently affected.

Previous studies by our group have shown that curcumin induces osteoblast apoptosis at doses of 12.5–25 μM and necrosis at doses greater than 50 μM [15]. Interestingly, curcumin is able to both stimulate and inhibit apoptotic signaling. For instance, curcumin induces apoptosis in human melanoma (30–60 μM for 24 h) [32], human leukemia HL 60 (10–40 μM for 16–24 h) [33,34], AK-5 tumor (10 μM for 18 h) [14,35] and MCF-7 breast cancer cells (25 μM for 24 h) [36]. Conversely, curcumin (10 μM for 12 h) inhibits dexamethasone-induced apoptosis in rat thymocytes, chemotherapy-induced apoptosis in breast cancer cells [37,38], and methylglyoxal-triggered apoptosis in mouse embryonic stem cells [21]. Our novel results, along with previously published findings, indicate that the treatment protocol (*i.e.*, treatment period and dosage) determines the effects of curcumin on various cell types. A recent study by our group further demonstrated that curcumin triggers apoptosis in ICM cells of mouse blastocysts, leading to impairment of embryo development via ROS generation, which in turn stimulates downstream processes through the mitochondria-dependent pathway [22]. In the present study, we further investigated whether curcumin exerts possible cytotoxic effects on oocyte maturation, fertilization, and sequential embryo development.

Oocyte maturation, fertilization, and embryonic development are complex processes during which chemical injury can lead to developmental problems or embryonic malformation. Previously, we showed that curcumin induces apoptosis, impairment of blastocyst development from the morula, and promotion of early-stage death of mouse blastocysts [22]. Thus, it is important to establish the possible teratogenic effects of curcumin. In this study, we demonstrate for the first time that curcumin inhibits mouse oocyte maturation, fertilization, and sequential embryonic development (Figure 1). Importantly, the curcumin-pretreated oocyte group displayed significantly decreased cell number and increased apoptosis (Figure 2A,B). The results collectively indicate that curcumin treatment at the oocyte stage triggers both oocyte maturation injury and abnormal apoptosis of cells at the blastocyst stage, an important step in embryo implantation.

The TE arises from the trophoblast at the blastocyst stage and develops into a sphere of epithelial cells surrounding the ICM and blastocoel. These cells contribute to the placenta and are required for mammalian conceptus development [39]. Reduction in cells of the TE and/or ICM lineage leads to suppressed implantation and lower embryonic viability [40,41]. The ICM and total blastocyst cell numbers are positively correlated with embryonic development during the embryo transfer assay [42]. Application of curcumin during oocyte maturation had no effect on the TE cell number of blastocysts, but led to a dramatic decrease in ICM and total (TE plus ICM) cell numbers (Figure 2). These results imply that curcumin treatment during IVM causes mortality and/or developmental delay in postimplantation mouse embryos via ICM cell death or a decrease in proliferation (Figures 2 and 3). Interestingly, blastocysts derived from curcumin-treated oocytes appeared to undergo decreased implantation, increased embryo resorption, and a lower fetal survival rate (Figure 3A), but exhibited comparable placental weight to the control group. TE cells of embryos play important roles in implantation and placental development. Our results indicate that curcumin does not exert a hazardous effect on TE cells of blastocysts and consequently does not affect placental development. Based on the data, we propose that the decrease in ICM cell number induced by curcumin during oocyte maturation is the major injurious factor leading to inhibition of embryonic development.

Our results show that curcumin-pretreated oocytes display significantly decreased fertilization rates and cleavage to the two-cell and blastocyst stages, compared to the untreated control groups (Figure 1), indicating that curcumin induces loss of fertilization and sequent embryonic development. Moreover, in an embryo transfer study, mouse blastocyst stage embryos derived from curcumin-pretreated oocytes displayed lower implantation and higher resorption rates than control blastocyst-derived untreated oocytes (Figure 3A). Further experiments disclosed lower embryo implantation rates and fetal weights and a higher resorption rate of blastocyst stage embryo transfer to the mouse uterus in the 40 μM curcumin-intake group, distinct from those of the curcumin-free control group (Figure 4A,B). These results collectively indicate that curcumin has potentially hazardous effects on early-stage oocyte maturation and fertilization. Importantly, exposure of blastocyst embryos to curcumin reduces the potential of embryo implantation and postimplantation development.

During embryonic development, cells are often poised between proliferation and apoptosis. Curcumin is cytotoxic to human osteoblasts and mouse blastocysts [15,22]. The compound induces apoptosis via activation of mitochondrial processes and caspases, which are the predominant pathways during oxidative stress-mediated cell injury [15,22]. Experiments from the present study showed that pretreatment of oocytes with curcumin leads to decreased cell number, apoptosis, and delay in postimplantation development of blastocysts, compared to the control group. These injurious effects were prevented by pretreatment of oocytes with a caspase-3 inhibitor to suppress blastocyst apoptosis (Figure 5). Treatment with the caspase-3 inhibitor additionally rescued curcumin-induced reduction in postimplantation development potential following embryo transfer, including embryo implantation rate, fetal survival rate, and fetal development status. Our results indicate that curcumin triggers improper cell apoptotic processes in early-stage embryos, leading to loss of embryo cell numbers and suppression of post-implantation development, further supporting its role as a teratogen through apoptosis induction in early-stage embryonic cells. Moreover, in the current study, we found that the levels of mRNA encoding p53 and p21 were upregulated in mouse blastocysts derived from oocytes pretreated with curcumin (Figure 6A). Further, siRNA-mediated knockdown of p53-encoding mRNA inhibited the curcumin-induced increase in the level of mRNA encoding p21 and decreased the extent of subsequent apoptosis (Figure 6A,B). These results indicate that p53 and p21 play apoptotic roles in mouse blastocyst cells derived from curcumin-treated oocytes.

## 3. Experimental Section

### 3.1. Chemicals and Reagents

Dulbecco’s modified Eagle’s medium (DMEM), curcumin, and pregnant mare serum gonadotropin (PMSG) were obtained from Sigma (St. Louis, MO, USA). Human chorionic gonadotropin (hCG) was purchased from Serono (NV Organon, Oss, The Netherlands). TUNEL *in situ* cell death detection kits were acquired from Roche (Mannheim, Germany), and CMRL-1066 medium from Gibco Life Technologies (Grand Island, NY, USA).

### 3.2. COC Collection and in Vitro Maturation (IVM)

ICR mice were acquired from the National Laboratory Animal Center (Taiwan). This research was approved by the Animal Research Ethics Board of Chung Yuan Christian University (Taiwan). All animals received humane care, as outlined in the Guidelines for Care and Use of Experimental Animals (Canadian Council on Animal Care, Ottawa, Canada, 1984). Mice were maintained on breeder chow (Harlan Teklad chow) with food and water available *ad libitum*. Housing was provided in standard 28 cm × 16 cm × 11 cm (height) polypropylene cages with wire-grid tops, and maintained under a 12 h day/12 h night regimen. Cumulus-oocyte complexes (COCs) were obtained according to a previous protocol [24]. Briefly, COCs were isolated from female hybrid ICR mice (21 days old) injected with 5 IU human chorionic gonadotrophin (hCG) 44 h prior to oocyte collection. COCs were collected in HEPES-buffered α minimum essential medium (MEM) (containing 50 μg/mL Streptomycin sulfate, 75 μg/mL Penicillin G, and 5% fetal bovine serum) by gently puncturing visible antral follicles present on the ovary surface. Germinal vesicle stage oocytes containing an intact vestment of cumulus cells were collected and pooled in at least 8 animals. For oocyte maturation, one drop (~100 μL) of buffer (α minimum essential medium supplemented with 50 μg/mL Streptomycin, 75 μg/mL Penicillin G, 5% FBS and 50 mIU/mL recombinant human FSH) containing 10 COCs was added under oil in 35 mm culture dishes. COC maturation was analyzed following treatment with or without various concentrations of curcumin (5, 10 or 20 μM) for 24 h under an atmosphere of 5% O_2_, 6% CO_2_ and balance of N_2_ at 37 °C.

### 3.3. Maturation Status Assessment

After *in vitro* maturation (IVM), COCs of each group were treated with 50 U/mL ovine hyaluronidase and gently pipetted for the removal of all cumulus cells. Denuded oocytes were collected, and washed with fresh medium, followed by phosphate-buffered saline (PBS). Oocytes were fixed in ethanol: glacial acetic acid (1:3) for 48 h, and stained with 1% aceto-orcein solution. Nuclear structures were visualized using phase-contrast microscopy.

### 3.4. *In Vivo* Maturation

For obtaining *in vivo* matured oocytes, 21 day-old mice were injected with 5 IU equine chorionic gonadotrophin (eCG) and 5 IU hCG, 61 and 13 h prior to fertilization, respectively. Mature ova were collected from the oviduct into HEPES-buffered α MEM medium.

### 3.5. Effects of Curcumin Intake on Oocyte Maturation in an Animal Model

The effects of curcumin on oocytes were analyzed in 21 day-old ICR virgin albino mice. Female mice were randomly divided into two groups of 20 animals each, and administered a standard diet with or without 10–40 μM curcumin in drinking water for 4 days. COCs were collected by pre-treatment with 5 IU human chorionic gonadotrophin (hCG) for 44 h prior to oocyte collection, and analyzed for oocyte maturation, *in vitro* fertilization, and embryonic development.

### 3.6. *In Vitro* Fertilization

For *in vitro* fertilization, ova were washed twice in bicarbonate-buffered α-MEM medium (containing 50 mg/mL Streptomycin, 75 mg/mL Penicillin G and 3 mg/mL fatty acid free bovine serum albumin), and fertilized in the same medium with fresh sperm (obtained from a CBAB6F1 male donor). After incubation with sperm for 4.5 h, eggs were washed three times in potassium simplex optimized medium (KSOM) without amino acids in the presence of l-alanyl- l-glutamine (1.0 mM). Next, eggs were placed in 20 mL drops of KSOM under oil, and cultured overnight. During cleavage to the 2-cell stage, embryos were transferred to a fresh drop of KSOM under oil, and cultured for another 72 h. All fertilization steps and embryo culture were additionally carried out under 5% O_2_, 6% CO_2_ and balance of N_2_ at 37 °C.

### 3.7. Fertilization Assessment

For the examination of fertilization, ova were incubated with sperm for 4.5 h, followed by 3 h of culture in fresh medium. Zygotes were assessed for the presence of the male pronucleus with orcein staining, as described previously [24].

### 3.8. Cell Proliferation

Cell proliferation was analyzed by dual differential staining, which facilitated the counting of cell numbers in inner cell mass (ICM) and trophectoderm (TE) [40,43,44]. Blastocysts were incubated with 0.4% pronase in M_2_–BSA medium (M_2_ medium containing 0.1% bovine serum albumin) for the removal of zona pellucida. Denuded blastocysts were exposed to 1 mM trinitrobenzenesulfonic acid (TNBS) in BSA-free M_2_ medium containing 0.1% polyvinylpyrrolidone (PVP) at 4 °C for 30 min, and washed with M_2_ [45]. Blastocysts were further treated with 30 μg/mL anti-dinitrophenol-BSA complex antibody in M_2_-BSA at 37 °C for 30 min, followed by M_2_ supplemented with 10% whole guinea pig serum as a source of complement, along with 20 μg/mL bisbenzimide and 10 μg/mL propidium iodide (PI) at 37 °C for 30 min. The immunolysed blastocysts were gently transferred to slides, and protected from light before observation. Under UV light, ICM cells (which take up bisbenzimidine but exclude PI) appeared blue, whereas TE cells (which take up both fluorochromes) appeared orange-red. Since multinucleated cells are not common in preimplantation embryos [46], the number of nuclei represent an accurate measurement of cell number.

### 3.9. TUNEL Assay of Blastocysts

For TUNEL staining, embryos were washed in curcumin-free medium, fixed, permeabilized, and subjected to labeling using an *in situ* cell death detection kit (Roche Molecular Biochemicals, Mannheim, Germany), according to the manufacturer’s protocol. Photographic images were obtained with a fluorescence microscope under bright-field illumination.

### 3.10. Blastocyst Development Following Embryo Transfer

To determine the ability of expanded blastocysts to implant and develop *in vivo*, embryos generated were transferred to recipient mice. ICR females (6–8 week-old, white skin) were mated with vasectomized males (C57BL/6J; black skin; National Laboratory Animal Center, Taiwan) to produce pseudopregnant dams as recipients for embryo transfer. To ensure that all fetuses in pseudopregnant mice were derived from embryo transfer (white color) and not fertilization by C57BL/6J (black color), we examined skin color at day 18 post-coitus. To assess the impact of curcumin on postimplantation growth *in vivo*, COCs were exposed to 0–20 μM curcumin for 24 h, followed by fertilization and *in vitro* maturation to the blastocyst stage. Subsequently, 8 untreated control embryos were transferred to the left uterine horn, and 8 curcumin-treated embryos to the right uterine horn in day 4 pseudopregnant mice. Forty surrogate mice were analyzed and killed on day 18 post-coitus, and the frequency of implantation calculated as the number of implantation sites per number of embryos transferred. The incidence rates of resorbed and surviving fetuses were calculated as number of fetuses per number of implantations, respectively. The weights of the surviving fetuses and placenta were measured immediately after dissection.

### 3.11. Statistical Analysis

Data were analyzed using one-way ANOVA and *t*-tests, and presented as means ± SEM. Data were considered statistically significant at *P* < 0.05.

## 4. Conclusions

Based on our study results, we propose that developmental injury by curcumin occurs via induction of apoptosis processes in oocyte maturation and early-stage embryos. We show for the first time that curcumin exerts injurious effects on oocyte maturation, fertilization, and embryonic development. Our findings clearly suggest that curcumin is a risk factor for normal embryonic development and possibly suppresses oocyte maturation in infertile couples. Further studies are required to ascertain the possible teratogenic actions of curcumin on human oocyte maturation and embryogenesis.
